# Evaluating the long-term impact of a distance learning course on attitudes, skills, practices, and knowledge in gender-affirming healthcare among healthcare professionals in Italy

**DOI:** 10.3389/fpubh.2025.1550470

**Published:** 2025-07-02

**Authors:** Matteo Marconi, Ughetta Maria Favazzi, Pietro Carbone, Debora Guerrera, Angela Ruocco, Martina Manoli, Cristiana Barbati, Francesca Molinaro, Federica Maria Regini, Andrea Vittozzi, Alfonso Mazzaccara, Marina Pierdominici

**Affiliations:** ^1^Reference Centre for Gender Medicine, Istituto Superiore di Sanità, Rome, Italy; ^2^Training Office, Istituto Superiore di Sanità, Rome, Italy; ^3^Department of Medical and Surgical Sciences and Advanced Technologies “GF Ingrassia”, University of Catania, Catania, Italy; ^4^Department of Infectious Diseases, Istituto Superiore di Sanità, Rome, Italy

**Keywords:** transgender and gender diverse, healthcare professionals, distance learning, continuing medical education, knowledge retention, gender-affirming care

## Abstract

**Introduction:**

Transgender and gender-diverse (TGD) individuals face significant health disparities, often due to healthcare providers’ (HCPs) insufficient training and awareness. Comprehensive educational interventions are essential to improve both cultural competence and medical knowledge. While previous evaluations of training programs have shown short-term benefits, evidence on long-term outcomes remains scarce. This study extends the findings of an earlier evaluation of a distance learning course on TGD healthcare, exploring the sustainability of its effects over time.

**Objectives:**

To assess changes in self-reported attitudes, skills, practices, and knowledge retention 6 months after course completion.

**Methods:**

A longitudinal design was used with assessments at baseline (T0), post-course (T1), and six-month follow-up (T2). Participants completed the Attitudes, Skills, and Practices Questionnaire (ASPQ) and a 10-item knowledge test at all time points. Paired *t*-tests were used to compare mean scores for attitudes and skills. Knowledge retention was analyzed with McNemar tests. Changes in practice items were evaluated using Cochran’s Q and Friedman tests for paired categorical data.

**Results:**

A total of 3,102 participants (17% of the original cohort) completed the follow-up. All self-reported skills and most attitudes improved significantly from baseline to follow-up (*p* < 0.001 for skills; *p* ≤ 0.02 for attitudes), with modest declines from T1. Practice items showed significant variation over time (Cochran’s Q and Friedman tests, *p* < 0.001), though without a consistent increase in engagement. Knowledge improved in 8 of 10 items from baseline to follow-up (McNemar, *p* < 0.001), particularly in sexual identity, hormone therapy, and legal rights, despite partial declines from post-course levels.

**Conclusion:**

This study highlights sustained improvements in self-reported attitudes, skills, and knowledge following a national TGD healthcare training, reinforcing its value in addressing health disparities. The findings underscore the need for structural support and integrated reinforcement to embed gender-affirming care into routine practice.

## 1 Introduction

Transgender and gender-diverse (TGD) individuals experience significant health disparities, often exacerbated by stigma, discrimination, and insufficient knowledge among healthcare professionals (HCPs) (1–4). Comprehensive training for HCPs is a crucial step in addressing these barriers and fostering equitable, gender-affirming care (5). Evidence suggests that educational interventions can positively impact HCPs’ knowledge, attitudes, and competencies in TGD healthcare. However, much of the existing research has focused on short-term outcomes, often measured immediately after training, with limited attention to the long-term retention of these improvements (6, 7). Longitudinal studies are essential to determine whether initial gains persist over time and translate into sustainable changes in clinical practice. The few available longitudinal evaluations of TGD health education for HCPs are often limited by small sample sizes or restricted to specific institutional settings (8–14), leaving a critical gap in the literature. This study builds upon our earlier work, which evaluated the impact of a 16-h, Continuing Medical Education (CME) accredited distance learning course on TGD health (15). The course encompassed a broad array of topics, including effective communication strategies, preventive care and lifestyle considerations, medical pathways for gender affirmation, and legal aspects of TGD healthcare. The initial results demonstrated significant improvements in participants’ knowledge and high levels of satisfaction. The current study examines six-month follow-up outcomes, focusing on self-reported attitudes, skills, practices, and knowledge retention, among over 3,000 participants. To the best of our knowledge, this investigation represents the largest longitudinal study in the field to date, providing valuable insights into the durability of training outcomes and the potential for large-scale educational interventions to drive systemic change in healthcare practices.

## 2 Materials and methods

### 2.1 Course design, learning methodology, and participant overview

The distance learning course, “*Transgender Population: From Health to Rights*,” was freely offered through the Istituto Superiore di Sanità (ISS)—National Institute of Health in Italy e-learning platform (EDUISS, www.eduiss.it) and delivered entirely in Italian. Details of this course have been published previously (15). It employed a Problem-Based Learning (PBL) framework, a methodology rooted in andragogic principles that fosters self-directed learning by engaging participants in solving real-world problems pertinent to their professional domains (16). The PBL methodology was adapted for e-learning, allowing participants to independently complete a structured seven-step cycle supported by interactive tools such as forums, quizzes, and curated resource materials.

A scientific board comprising experts in TGD healthcare, legal scholars, and representatives from TGD advocacy organizations designed this pioneering course. The curriculum systematically addressed critical areas of TGD care, including:

- Biological underpinnings of sexual identity.
- Psychological support spanning developmental and adult stages.
- Evidence-based approaches to hormone therapy.
- Core principles and practices in gender-affirmation surgery.
- Legal frameworks governing gender identity rights in Italy.

These topics were distilled into five targeted Learning Objectives (LOs). The course was accessible to all healthcare professionals and ran from March 27 to September 22, 2023, with a participant capacity of up to 30,000. Upon successful completion, participants earned 16 CME credits, requiring approximately 16 h of engagement. In accordance with current Italian and European Union regulations, this type of non-interventional educational study did not require formal approval by an ethics committee. Participation was voluntary, and informed consent for the use of pseudonymized data was obtained from all participants at the time of course registration.

The course was systematically divided into four sections:

1. *Introductory section*

- This section provided a comprehensive overview, outlining the course’s relevance, objectives, structure, and intended goals. Participants received detailed guidelines to facilitate their participation.
- It included the Attitudes, Skills, and Practices Questionnaire (ASPQ) at baseline (T0) and a Multiple Choice Question (MCQ) knowledge assessment test to assess prior knowledge (pre-test). No minimum score was required for pre-test completion.

2. *PBL cycle*

This section provided an array of resources to support the PBL cycle. These included problem scenarios, a Sharable Content Object Reference Model (SCORM) exercise for problem analysis and identification of LOs, research materials (e.g., bibliographic references, recommended websites, specialized readings, and expert-led audio-video tutorials), and activities for problem resolution.

3. *Concluding section*

- Participants completed the ASPQ at T1 (immediate post-course) and the same MCQ knowledge assessment test used in the pre-test to evaluate knowledge acquisition (post-test). This section concluded with a final certification exam and an optional Satisfaction Questionnaire (SQ).
- The SQ was available exclusively to participants who finished all learning units. To qualify for CME credits, participants were required to pass the final exam, which consisted of 48 MCQs and required a minimum passing score of 75%. A maximum of three attempts was permitted.

4. *Follow-up section*

Six months post-course (T2), participants completed the follow-up administration of the ASPQ and repeated the same MCQ knowledge assessment test used at T0 and T1. Both were available from March 26 to May 20, 2024.

### 2.2 Data collection and tools

As part of the enrollment process for the advanced educational course hosted on the ISS e-learning platform, participants were required to provide demographic and professional details, including gender, age, geographic location, CME profession, and discipline. Two key tools were used throughout the study to establish a baseline and monitor progress over time:

1. ASPQ: the ASPQ is a purpose-built instrument developed specifically for this study, aligned with the course objectives. It was administered at three time points—T0 (baseline), T1 (immediate post-course), and T2 (six-month follow-up)—to assess changes over time. It consists of item batteries using Likert-scale formats across several domains:

- Attitudes: five items rated on a 5-point Likert scale
- Skills: five items rated on a 5-point Likert scale
- Practices: four items, combining dichotomous and polytomous response formats

2. Knowledge assessment test: this test comprises 10 MCQs (two items per LO), designed to evaluate core conceptual knowledge aligned with the specific objectives of the course. The same version of this test was administered at three time points:

- T0 (Pre-test): to establish a baseline prior to course access
- T1 (Post-test): immediately after course completion, to assess knowledge acquisition
- T2 (Follow-up): 6 months after course completion, to assess long-term knowledge retention

The SQ, administered only at T1, includes 18 items on a 5-point Likert scale and two open-ended questions aimed at evaluating course satisfaction and collecting suggestions for improvement.

The results of the knowledge assessment test at T0 and T1, as well as those of the SQ have been published previously (15). The present analysis focused on participants who successfully completed the course, passed the final certification, and voluntarily participated in the six-month follow-up phase. This cohort consisted of 3,102 individuals who completed both follow-up instruments (ASPQ and the knowledge assessment test), ensuring a robust dataset for longitudinal analysis.

### 2.3 Statistical analysis

All data were extracted from the EDUISS platform. A descriptive analysis, including absolute numbers and percentages, was performed to present demographic and professional characteristics.

To assess the psychometric properties of the Attitudes and Skills sections of the ASPQ, an Exploratory Factor Analysis (EFA) was performed at T0, T1, and T2 using principal axis factoring with Promax rotation. The analysis consistently revealed a two-factor structure aligned with the theoretical constructs of self-perceived skills (Items 1–5) and attitudes (Items 6–10). Internal consistency was evaluated using Cronbach’s alpha, which indicated high reliability across all three time points (Skills: *α* = 0.897–0.914; Attitudes: *α* = 0.862–0.918).

The Practices section was excluded from these psychometric analyses because it consists of one dichotomous item and three ordinal items with five response options. Due to their categorical nature and heterogeneity, these items were analyzed descriptively.

The Likert-scale data from the ASPQ, focusing on Attitudes and Skills, were analyzed using two approaches: (1) frequency distributions of responses were examined at T0, T1, and T2; (2) paired *t*-tests were conducted to compare mean scores between T0 and T2 and T1 and T2, to identify statistically significant differences. Changes in responses to the Practices items over time were assessed using non-parametric tests suitable for paired data. For the dichotomous item assessing whether participants had ever provided care to TGD individuals, Cochran’s Q test was used to assess differences across T0, T1, and T2. For the three ordinal items related to family engagement and the provision of information on gender-affirming care and legal aspects, Friedman tests were conducted to evaluate changes in response distributions over time. Results from the knowledge assessment test at T0, T1 and T2 were expressed as the percentage of correct answers for each question and compared using the McNemar’s test.

All statistical analyses were performed using IBM SPSS Statistics version 28.0. Statistical significance was set at *p* < 0.05.

## 3 Results

### 3.1 Characteristics of participants

The study population refers to a broader group of 18,282 individuals who completed the course, as described in a previous study (15). The current analysis included 3,102 healthcare professionals. The majority of participants were female (73.9%), while 26.1% were male. The participants’ mean age was 49.6 years (SD 10.3), and the age group most represented was 46 to 55 years (38.5%), followed by those over 55 years (32.1%). Younger professionals, specifically those up to 35 years, accounted for only 13.1% of the sample.

In terms of geographical distribution, the Northwest region of Italy had the highest participation, with 35.4% of individuals coming from this area. The Center followed with 23.9%, while the South and the Islands accounted for 15.6 and 10.0%, respectively. Professionally, the participants came from a variety of healthcare roles. Nurses represented 47.8% of the sample, making them the largest professional group among the participants. These results are reported in Table 1.

**Table 1:** Characteristics of participants.

Characteristic	*N* (%)
Gender	
Male	811 (26.1)
Female	2,291 (73.9)
Age (years)	
Up to 35 years	406 (13.1)
36 to 45 years	504 (16.3)
46 to 55 years	1,194 (38.5)
Over 55 years	997 (32.1)
Italian region area	
Northwest	1,099 (35.4)
Northeast	462 (14.9)
Center	741 (23.9)
South	485 (15.6)
Islands	311 (10.0)
Abroad	4 (0.1)
Health professions	
Medical doctor	199 (6.4)
Dentist	15 (0.5)
Pharmacist	40 (1.3)
Veterinarian	4 (0.1)
Psychologist	236 (7.6)
Biologist	84 (2.7)
Chemist	37 (1.2)
Physicist	7 (0.2)
Rehabilitation professions	479 (15.4)
Professional educator	138 (4.4)
Physiotherapist	262 (8.4)
Speech therapist	35 (1.1)
Orthoptist/ophthalmology assistant	12 (0.4)
Podiatrist	1 (0.0)
Psychiatric rehabilitation technician	13 (0.4)
Developmental neuro and psychomotor therapist	8 (0.3)
Occupational therapist	10 (0.3)
Preventive health professions	63 (2.0)
Healthcare assistant	31 (1.0)
Environmental and workplace prevention technician	32 (1.0)
Nursing profession	
Nurse	1,484 (47.8)
Midwifery profession	
Midwife	44 (1.4)
Technical health professions—technical assistance area	39 (1.3)
Dietitian	14 (0.5)
Dental hygienist	9 (0.3)
Hearing care technician	10 (0.3)
Cardiovascular pathophysiology and perfusion technician	5 (0.2)
Orthopedic technician	1 (0.0)
Technical health professions—diagnostic area	371 (12.0)
Audiometrist technician	7 (0.2)
Neurophysiopathology technician	14 (0.5)
Medical radiology technician	138 (4.4)
Biomedical laboratory health technician	212 (6.8)
Overall	3,102 (100)

The table reports the characteristics of course participants, including gender, age, Italian regional area, and health profession. For clarity regarding the listed health professions, it should be noted that in the Italian healthcare system, the category of “health professions” may include not only clinical roles (such as physicians and nurses), but also chemists and physicists engaged in clinical or diagnostic activities.

### 3.2 Changes in self-reported attitudes

The results, presented in Table 2 as mean scores and in Supplementary Table 1 as percentage distributions across the Likert scale, indicate significant changes over time in participants’ beliefs regarding the care of TGD individuals.

**Table 2:** Self-reported attitudes toward the care of TGD individuals.

Statement	T0	T1	T2	T2–T0	*p*-Value	T2–T1	*p*-Value
With regard to the care of individuals experiencing gender incongruence, I believe that:							
The role of biological factors in the development of gender differences is significant	3.53	3.88	3.65	0.12	<0.001	−0.23	<0.001
Psychological support should be provided, if requested, during one or more phases of the gender-affirming pathway	4.35	4.33	4.39	0.03	0.020	−0.06	<0.001
Information on the available options for gender-affirming hormonal therapy should be provided	4.24	4.33	4.33	0.09	<0.001	0.00	0.925
Appropriate information on the fundamental aspects of gender-affirming surgical procedures should be made available	4.29	4.36	4.39	0.09	<0.001	−0.02	0.066
Information regarding the general principles of the right to gender identity under Italian law should be offered	4.29	4.35	4.34	0.06	<0.001	−0.01	0.642

The number of participants who completed the follow-up was 3,102. Data were analyzed using paired *t*-test. T0, baseline assessment; T1, immediate post-course assessment; T2, six-month follow-up assessment.

Regarding the belief that the role of biological factors in the development of gender differences is relevant, the mean score increased from 3.53 at T0 to 3.88 at T1 and slightly decreased to 3.65 at T2, with a mean difference of 0.12 between T0 and T2 (*p* < 0.001). For the belief that psychological support should be provided, if required, during one or more stages of the gender-affirming treatment, scores remained high, increasing slightly from 4.35 at T0 to 4.39 at T2, with a mean difference of 0.03 between T0 and T2 (*p* = 0.020).

The belief that individuals should be informed on possible gender-affirming hormone therapy options showed a slight increase from 4.24 at T0 to 4.33 at T1 and T2, with a mean difference of 0.09 between T0 and T2 (*p* < 0.001). Similarly, for the belief that appropriate information should be provided on the key aspects of gender-affirming surgery, the mean score increased from 4.29 at T0 to 4.36 at T1 and further to 4.39 at T2, with a mean difference of 0.09 between T0 and T2 (*p* < 0.001). Finally, regarding the belief that information should be provided on the general principles of the right to gender identity in Italian law, the mean score increased slightly from 4.29 at T0 to 4.35 at T1 and slightly decreased to 4.34 at T2, with a mean difference of 0.06 between T0 and T2 (*p* < 0.001). In summary, the findings demonstrate meaningful changes in beliefs regarding the care of TGD individuals, with small but statistically significant differences observed between baseline (T0) and follow-up (T2). Comparisons between T1 and T2 showed minimal or small variations, some of which reached statistical significance but were limited in magnitude, indicating that attitudes were generally sustained over time.

### 3.3 Changes in self-reported skills

The results, presented in Table 3 as mean scores and in Supplementary Table 2 as percentage distributions across the Likert scale, indicate significant changes over time in participants’ self-reported capabilities. Regarding the ability to describe the components of sexual identity, the mean score increased from 2.70 at T0 to 3.72 at T1, then slightly declined to 3.35 at T2, with a mean difference of 1.02 between T0 and T2 (*p* < 0.001). For the ability to recognize key aspects relevant to psychological support during both developmental stages and adulthood, the mean score rose from 2.70 at T0 to 3.77 at T1 and slightly decreased to 3.49 at T2, with a mean difference of 0.79 between T0 and T2 (*p* < 0.001). The ability to identify best practices for gender-affirming hormonal treatment across developmental stages and adulthood showed an increase in mean scores from 2.20 at T0 to 3.55 at T1, followed by a slight decline to 3.14 at T2, with a mean difference of 0.94 between T0 and T2 (*p* < 0.001). Similarly, the ability to define the fundamental principles underlying gender-affirming surgery rose from 2.18 at T0 to 3.61 at T1 and decreased to 3.24 at T2, with a mean difference of 1.06 between T0 and T2 (*p* < 0.001). Lastly, for the ability to outline the general principles governing the right to gender identity within the framework of Italian law, the mean score increased from 2.40 at T0 to 3.77 at T1, then slightly decreased to 3.37 at T2, with a mean difference of 0.98 between T0 and T2 (*p* < 0.001).

**Table 3:** Self-reported skills in the care of TGD individuals.

Statement	T0	T1	T2	T2-T0	*p*-Value	T2-T1	*p*-Value
With regard to the care of individuals experiencing gender incongruence, if given the opportunity, I would be able to:							
Describe the components of sexual identity	2.70	3.72	3.35	1.02	<0.001	−0.37	<0.001
Recognize key aspects relevant to psychological support during both developmental stages and adulthood	2.70	3.77	3.49	0.79	<0.001	−0.28	<0.001
Identify best practices for hormonal treatment across developmental stages and adulthood	2.20	3.55	3.14	0.94	<0.001	−0.41	<0.001
Define the fundamental principles underlying gender-affirming surgery	2.18	3.61	3.24	1.06	<0.001	−0.37	<0.001
Outline the general principles governing the right to gender identity within the framework of Italian law	2.40	3.77	3.37	0.98	<0.001	−0.40	<0.001

The number of participants who completed the follow-up was 3,102. Data were analyzed using paired *t*-test. T0, baseline assessment; T1, immediate post-course assessment; T2, six-month follow-up assessment.

In summary, while all capabilities demonstrated a statistically significant decline at T2 compared to immediate post-course scores (T1), mean scores remained substantially higher than baseline (T0), indicating sustained improvement over time. These findings suggest that, despite some attenuation, self-reported competencies were meaningfully retained 6 months after course completion.

### 3.4 Changes in self-reported practices

The results, summarized in Table 4, indicate that the direct management of gender-affirming care remains a relatively uncommon component of clinical practice for most participants. Statistically significant variations over time were observed across the four self-reported practices assessed, although these did not uniformly reflect increased engagement. The proportion of respondents who reported having provided care to TGD individuals decreased from 33.0% at T0 to 31.7% at T1 and 28.2% at T2 (*p* < 0.001), possibly reflecting a more cautious or accurate interpretation of what constitutes gender-specific care following the training intervention. Responses concerning interactions with the families of TGD individuals changed significantly over time (*p* < 0.001), as did the frequency of providing information on gender-affirming pathways (*p* < 0.001) and on legal aspects of gender-affirming care (*p* < 0.001). These shifts in response distributions were statistically significant, but no consistent directional trend (e.g., toward more frequent engagement) was observed across time points.

**Table 4:** Self-reported practices regarding TGD individuals.

Survey item	T0 (%)	T1 (%)	T2 (%)	Statistical test	*p*-Value
Have you ever provided care to TGD individuals in your professional practice?					
Yes	1,025 (33.0)	983 (31.7)	874 (28.2)	Cochran’s Q	<0.001
No	2077 (67.0)	2,119 (68.3)	2,228 (71.8)		
Overall	100.0	100.0	100.0		
I interact with the families of TGD individuals					
Never	894 (28.8)	886 (28.6)	981 (31.6)	Friedman	<0.001
Rarely	1,043 (33.6)	1,129 (36.4)	1,078 (34.8)		
Sometimes	739 (23.8)	694 (22.4)	674 (21.7)		
Often	78 (2.5)	105 (3.4)	88 (2.8)		
Regularly	37 (1.2)	49 (1.6)	20 (0.6)		
I have never provided care to TGD individuals	311 (9.9)	239 (7.7)	261 (8.4)		
In my professional activity, I provide information on gender-affirming care pathways					
Never	1,522 (49.1)	1,366 (44.0)	1,504 (48.5)	Friedman	<0.001
Rarely	833 (26.9)	912 (29.4)	817 (26.3)		
Sometimes	382 (12.3)	477 (15.4)	459 (14.8)		
Often	73 (2.4)	72 (2.3)	77 (2.5)		
Regularly	23 (0.7)	42 (1.4)	22 (0.7)		
I have never provided care to TGD individuals	269 (8.7)	233 (7.4)	223 (7.2)		
In my professional activity, I provide information on legal issues related to gender-affirming pathways					
Never	1821 (58.8)	1,496 (48.2)	1,664 (53.6)	Friedman	<0.001
Rarely	653 (21.0)	857 (27.6)	756 (24.4)		
Sometimes	288 (9.3)	413 (13.3)	382 (12.3)		
Often	41 (1.4)	71 (2.3)	64 (2.1)		
Regularly	27 (0.9)	31 (1.0)	18 (0.6)		
I have never provided care to TGD individuals	272 (8.6)	234 (7.5)	218 (7.0)		

The number of participants who completed the follow-up was 3,102. T0, baseline assessment; T1, immediate post-course assessment; T2, six-month follow-up assessment.

### 3.5 Knowledge retention

The results, presented in Table 5 as percentages of correct answers at T0, T1, and T2, indicate significant changes in participants’ knowledge across all LOs.

**Table 5:** Knowledge assessment test: retention over time.

Learning Objective with corresponding assessment item		Correct answers	Correct answers	Correct answers	T2–T0	*p*-Value	T2–T1	*p*-Value
		T0 (%)	T1 (%)	T2 (%)				
LO1—describe the components of sexual identity and its biological bases	Q1	1,229 (39.6)	1848 (59.6)	1,534 (49.5)	9.9	<0.001	−10.1	<0.001
	Q2	843 (27.2)	1,392 (44.9)	967 (31.2)	4.0	<0.001	−13,7	<0.001
LO2—identify the aspects useful for providing psychological support to individuals experiencing gender incongruence, both in developmental stages and adulthood	Q3	1817 (58.6)	2,264 (73.0)	2052 (66.2)	7.6	<0.001	−6.8	<0.001
	Q4	2,124 (68.5)	2,218 (71.5)	2094 (67.5)	−1.0	0.363	−4.0	<0.001
LO3—identify best practices for gender-affirming hormone therapy, spanning across developmental stages and adulthood	Q5	1,399 (45.1)	2045 (65.9)	1,597 (51.5)	6.4	<0.001	−14.4	<0.001
	Q6	495 (16.0)	1,167 (37.6)	735 (23.7)	7.7	<0.001	−13.9	<0.001
LO4—outline the fundamental aspects of gender-affirming surgery	Q7	2,378 (76.7)	2,492 (80.3)	2,406 (77.6)	0.9	0.380	−2.7	0.004
	Q8	2,746 (88.5)	2,720 (87.7)	2,645 (85.3)	−3.2	<0.001	−2.4	0.003
LO5—understand the general principles of gender identity rights according to Italian law	Q9	914 (29.5)	2,199 (70.9)	1,419 (45.7)	16.2	<0.001	−25.2	<0.001
	Q10	1,094 (35.3)	1,541 (49.7)	1,489 (48.0)	12.7	<0.001	−1.7	0.155

The number of participants who completed the follow-up was 3,102. Data were compared through the McNemar test. LO, Learning Objective; T0, baseline assessment (pre-test); T1, immediate post-course assessment (post-test); T2, six-month follow-up assessment (Follow-up); Q, question.

For LO1: *Describe the components of sexual identity and its biological bases*, question Q1 showed an increase in correct responses from 39.6% at T0 to 59.6% at T1, followed by a decrease to 49.5% at T2, yielding a net gain of 9.9% between T0 and T2 (*p* < 0.001). Similarly, Q2 increased from 27.2% at T0 to 44.9% at T1, then decreased to 31.2% at T2, with a net gain of 4.0% between T0 and T2 (*p* < 0.001).

For LO2: *Identify the aspects useful for providing psychological support*, Q3 rose from 58.6% at T0 to 73.0% at T1, followed by a decline to 66.2% at T2, reflecting a net gain of 7.6% between T0 and T2 (*p* < 0.001). In contrast, Q4 showed minimal change, increasing from 68.5% at T0 to 71.5% at T1 and declining slightly to 67.5% at T2, with no significant difference between T0 and T2 (*p* = 0.363).

For LO3: *Identify best practices for gender-affirming hormone therapy,* Q5 increased from 45.1% at T0 to 65.9% at T1 before declining to 51.5% at T2, resulting in a net gain of 6.4% (*p* < 0.001). Q6 showed more notable improvement, rising from 16.0% at T0 to 37.6% at T1 and stabilizing at 23.7% at T2, with a net gain of 7.7% (*p* < 0.001).

For LO4: *Outline the fundamental aspects of gender-affirming surger*y, Q7 demonstrated stable performance, slightly increasing from 76.7% at T0 to 80.3% at T1 and returning to 77.6% at T2, with no significant difference between T0 and T2 (*p* = 0.380). Q8, however, showed a modest decline from 88.5% at T0 to 87.7% at T1 and further to 85.3% at T2, resulting in a net decrease of 3.2% (*p* < 0.001).

Finally, for LO5: *Understand the general principles of gender identity rights according to Italian law*, Q9 exhibited substantial improvement, increasing from 29.5% at T0 to 70.9% at T1 and stabilizing at 45.7% at T2, resulting in a net gain of 16.2% (*p* < 0.001). Similarly, Q10 rose from 35.3% at T0 to 49.7% at T1, with a slight decline to 48.0% at T2, resulting in a net gain of 12.7% (*p* < 0.001).

In summary, the results highlight net improvements across most LOs at T2 compared to T0. Notable gains were sustained for LO1, LO3, and LO5, while LO2 and LO4 showed minimal or no significant changes. Although a partial decline in knowledge retention was observed between T2 and immediate post-course performance (T1), overall knowledge at T2 remained markedly higher than at baseline.

## 4 Discussion

This study builds upon the work of Favazzi et al. (15), which demonstrated significant short-term improvements in HCPs’ knowledge following a distance learning program on gender-affirming care. By evaluating outcomes 6 months post-training, it provides a unique perspective on the sustainability of these improvements, with a focus on self-reported attitudes, skills, practices, and knowledge retention among a large cohort of over 3,000 participants.

Previous research has consistently highlighted the potential of educational interventions to enhance HCPs’ competence in TGD care. Short-term follow-up studies—typically conducted within 3 months of training—have demonstrated marked improvements in knowledge, comfort, and perceived confidence (11–14). The few available long-term evaluations, often conducted with small cohorts, have underscored the benefits of targeted training while emphasizing the necessity of ongoing reinforcement to maintain progress (9, 10, 17, 18). For example, Zheng et al. (10) reported sustained but gradually declining knowledge and confidence 1 year after a training session, underscoring the well-documented need for periodic refreshers.

Our study distinguishes itself in several significant ways. First, it represents, to our knowledge, the largest longitudinal cohort to date assessing the outcomes of a structured, institutionalized educational program in TGD healthcare. Second, while much of the existing literature is US-centric, this study provides crucial data from a European context, broadening the geographical scope of evidence. Third, the program’s inclusive design—targeting a wide range of HCPs—enhances its generalizability and applicability across diverse professional settings.

The analysis of attitudes reflects progress and challenges in fostering enduring change. Attitudes toward the importance of psychological support and the provision of information on gender-affirming treatments were consistently high, suggesting that participants already valued these aspects or that the training reinforced pre-existing beliefs. However, limited attitudinal shifts over time may indicate a need for more experiential learning approaches, such as role-playing or direct interaction with TGD individuals, to deepen empathy and internalize these principles more effectively. Such methods could move participants from abstract agreement to active, patient-centered practice (18).

Self-reported skills—such as the ability to describe the components of sexual identity, recognize psychological support needs, and identify best practices for gender-affirming medical and surgical care— also showed substantial gains from baseline (T0) to immediate post-training (T1) and remained meaningfully elevated at six-month follow-up (T2), despite a modest decline relative to T1. This pattern aligns with cognitive theories such as Ebbinghaus’ forgetting curve (19), confirming that without reinforcement, skills and knowledge naturally attenuate over time. Nevertheless, the persistence of improved competencies at T2 demonstrates the program’s efficacy in fostering durable learning.

The practices domain warrants particular attention. Consistent with prior literature highlighting HCPs’ limited exposure to TGD individuals (6), our findings confirm that direct management of gender-affirming pathways remains relatively infrequent. While the overall proportion of participants who reported providing direct care to TGD individuals declined slightly over time, significant shifts were noted in behaviors such as engaging with families and disseminating information about gender-affirming pathways and legal rights. These changes suggest that, despite limited direct clinical engagement—often constrained by structural factors—the training influenced broader aspects of care delivery. Notably, the slight reduction in reported direct care may reflect a more refined understanding of gender-specific clinical roles following the training. This underscores the critical need for institutional and systemic support to translate individual competencies into routine practice. Future initiatives should prioritize not only education at the individual level but also organizational strategies—such as clear protocols and supportive infrastructures—to enable sustained integration of gender-affirming care across healthcare settings.

Finally, knowledge retention varied across LOs. Gains were particularly notable in understanding the biological foundations of sexual identity and the legal frameworks governing gender identity rights in Italy. Although knowledge related to psychological support and gender-affirming surgery showed less pronounced change—likely reflecting high baseline familiarity or potential limitations in the complexity of assessment items—overall performance at T2 remained markedly higher than at T0. Revising the assessment tools to incorporate greater complexity and better reflect the multifaceted realities of clinical practice could further enhance the accuracy of progress evaluations and the overall impact of the training.

Despite its strengths, this study is not without limitations. The reliance on self-reported measures to assess attitudes and skills, and practices while valuable, may not fully capture real-world clinical behaviors or patient outcomes. Additionally, the reduction in participant numbers at the six-month follow-up, though still yielding a robust sample, raises the possibility of selection bias, as those who completed the follow-up may represent the most motivated or engaged participants. This could lead to an overestimation of the program’s overall impact. The absence of a control group further limits the ability to isolate the program’s effects from external factors, such as exposure to other educational resources. Another important limitation concerns the ASPQ tool itself, which, although internally validated through exploratory factor analysis and internal consistency testing, remains a novel instrument that has not yet undergone external validation. While this limits the generalizability of the findings and calls for cautious interpretation, it may nonetheless serve as a useful foundation for future research and practice aimed at strengthening the assessment of competencies in gender-affirming care. Finally, the design of some assessment items may have contributed to high baseline scores in certain areas, necessitating revisions to improve their discriminatory power and ensure a more comprehensive evaluation of knowledge acquisition.

Taken together, the findings demonstrate that the program was effective in enhancing self-reported attitudes, skills, and knowledge among participants over the long term. Sustained improvements will likely require the integration of periodic reinforcement strategies to consolidate learning and promote the consistent delivery of gender-affirming care in clinical practice.

## 5 Conclusion

This study offers valuable insights into the long-term outcomes of a national educational initiative on TGD healthcare in Italy. By demonstrating sustained improvements in self-reported attitudes, skills, and knowledge, it provides a strong foundation for addressing healthcare disparities affecting TGD individuals. Although self-reported practices showed significant shifts over time, the results were heterogeneous, underscoring the importance of structural and organizational support in translating individual learning into consistent clinical practice. Moving forward, expanding and refining such programs—coupled with institutional policies and integrated reinforcement strategies—will be crucial for embedding gender-affirming care into routine healthcare delivery and advancing equity both nationally and internationally.

## Data Availability

The raw data supporting the conclusions of this article will be made available by the authors, without undue reservation.
