# Supplementary material for: Evaluating the long-term impact of a distance learning course on attitudes, skills, practices, and knowledge in gender-affirming healthcare among healthcare professionals in Italy
Source: Front Public Health. 2025 Jul 2;13:1550470. doi: 10.3389/fpubh.2025.1550470 (PMC12265919; doi:10.3389/fpubh.2025.1550470)
Supplement: Supplementary file 1 [file Supplementary_file_1.docx]

**Supplementary Table 1.** Self-Reported attitudes toward the care of TGD individuals.

|  | T0 (%) | T1 (%) | T2 (%) |
| --- | --- | --- | --- |
| **With regard to the care of individuals experiencing gender incongruence, I believe that:** |  |  |  |
| The role of biological factors in the development of gender differences is significant |  |  |  |
| Strongly disagree | 1.6 | 0.6 | 1.2 |
| Disagree | 6.2 | 1.9 | 5.0 |
| Neither agree nor disagree | 39.1 | 23.5 | 32.7 |
| Agree | 44.0 | 56.7 | 49.2 |
| Strongly agree | 9.1 | 17.3 | 11.9 |
| Psychological support should be provided, if requested, during one or more phases of the gender-affirming pathway |  |  |  |
| Strongly disagree | 0.9 | 0.5 | 0.5 |
| Disagree | 0.4 | 0.3 | 0.4 |
| Neither agree nor disagree | 10.4 | 10.2 | 10.2 |
| Agree | 39.4 | 43.8 | 37.8 |
| Strongly agree | 49.0 | 45.2 | 51.1 |
| Information on the available options for gender-affirming hormonal therapy should be provided |  |  |  |
| Strongly disagree | 0.7 | 0.5 | 0.4 |
| Disagree | 0.3 | 0.2 | 0.4 |
| Neither agree nor disagree | 11.8 | 9.5 | 10.5 |
| Agree | 43.2 | 45.2 | 43.2 |
| Strongly agree | 43.9 | 44.6 | 45.5 |
| Appropriate information on the fundamental aspects of gender-affirming surgical procedures should be made available |  |  |  |
| Strongly disagree | 0.7 | 0.4 | 0.3 |
| Disagree | 0.3 | 0.1 | 0.4 |
| Neither agree nor disagree | 11.8 | 9.7 | 10.1 |
| Agree | 43.2 | 42.5 | 39.1 |
| Strongly agree | 43.8 | 47.3 | 50.2 |
| Information regarding the general principles of the right to gender identity under Italian law should be offered |  |  |  |
| Strongly disagree | 0.7 | 0.4 | 0.4 |
| Disagree | 0.2 | 0.2 | 0.4 |
| Neither agree nor disagree | 11.6 | 9.3 | 10.6 |
| Agree | 44.8 | 44.1 | 41.7 |
| Strongly agree | 42.7 | 45.9 | 46.9 |

The number of participants who completed the follow-up was 3,102. T0, pre-test; T1, post-test; T2, six months follow-up test.

**Supplementary Table 2.** Self-Reported skills in the care of TGD individuals.

|  | T0 (%) | T1 (%) | T2 (%) |
| --- | --- | --- | --- |
| **With regard to the care of individuals experiencing gender incongruence, if given the opportunity, I would be able to:** |  |  |  |
| Describe the components of sexual identity |  |  |  |
| Strongly disagree | 8.3 | 0.5 | 1.4 |
| Disagree | 32.4 | 4.1 | 12.0 |
| Neither agree nor disagree | 43.3 | 29.7 | 43.6 |
| Agree | 13.2 | 54.1 | 36.0 |
| Strongly agree | 2.8 | 11.6 | 7.0 |
| Recognize key aspects relevant to psychological support during both developmental stages and adulthood |  |  |  |
| Strongly disagree | 8.8 | 0.7 | 1.3 |
| Disagree | 34.5 | 3.8 | 9.3 |
| Neither agree nor disagree | 37.4 | 26.4 | 36.8 |
| Agree | 16.1 | 56.4 | 44.6 |
| Strongly agree | 3.2 | 12.7 | 8.1 |
| Identify best practices for hormonal treatment across developmental stages and adulthood |  |  |  |
| Strongly disagree | 30.9 | 1.7 | 5.3 |
| Disagree | 31.3 | 7.6 | 17.5 |
| Neither agree nor disagree | 26.8 | 34.3 | 41.5 |
| Agree | 8.5 | 47.5 | 29.1 |
| Strongly agree | 2.5 | 9.0 | 6.6 |
| Define the fundamental principles underlying gender-affirming surgery |  |  |  |
| Strongly disagree | 31.1 | 1.6 | 3.4 |
| Disagree | 31.7 | 6.2 | 15.1 |
| Neither agree nor disagree | 27.0 | 32.3 | 42.3 |
| Agree | 8.3 | 49.9 | 32.8 |
| Strongly agree | 1.9 | 10.1 | 6.3 |
| Outline the general principles governing the right to gender identity within the framework of Italian law |  |  |  |
| Strongly disagree | 20.3 | 0.8 | 2.2 |
| Disagree | 35.6 | 3.2 | 11.3 |
| Neither agree nor disagree | 30.8 | 27.3 | 41.3 |
| Agree | 10.5 | 55.7 | 37.3 |
| Strongly agree | 2.7 | 13.1 | 7.9 |

The number of participants who completed the follow-up was 3,102. T0, pre-test; T1, post-test; T2, six months follow-up test.
